# A Safe and Effective Magnetic Labeling Protocol for MRI-Based Tracking of Human Adult Neural Stem Cells

**DOI:** 10.3389/fnins.2019.01092

**Published:** 2019-10-11

**Authors:** Albrecht Stroh, Jenny Kressel, Roland Coras, Antje Y. Dreyer, Wenke Fröhlich, Annette Förschler, Donald Lobsien, Ingmar Blümcke, Saida Zoubaa, Jürgen Schlegel, Claus Zimmer, Johannes Boltze

**Affiliations:** ^1^Institute for Pathophysiology, Mainz University, Mainz, Germany; ^2^German Resilience Center, Mainz, Germany; ^3^Department of Neuroradiology, Technical University Munich, Munich, Germany; ^4^Helmholtz Center Munich, Institute for Biological and Medical Imaging, Munich, Germany; ^5^Department of Neuropathology, University Hospital Erlangen, Erlangen, Germany; ^6^Translational Center for Regenerative Medicine, Fraunhofer Institute for Cell Therapy and Immunology, University of Leipzig, Leipzig, Germany; ^7^Department of Neuroradiology, University Hospital Leipzig, Leipzig, Germany; ^8^Division of Neuropathology, Institute of Pathology, Technical University of Munich, Munich, Germany; ^9^School of Life Sciences, University of Warwick, Coventry, United Kingdom

**Keywords:** human adult stem cells, magnetic labeling, MRI, cell tracking, CNS – disorder

## Abstract

Magnetic resonance imaging (MRI) provides a unique tool for *in vivo* visualization and tracking of stem cells in the brain. This is of particular importance when assessing safety of experimental cell treatments in the preclinical or clinical setup. Yet, specific imaging requires an efficient and non-perturbing cellular magnetic labeling which precludes adverse effects of the tag, e.g., the impact of iron-oxide-nanoparticles on the critical differentiation and integration processes of the respective stem cell population investigated. In this study we investigated the effects of very small superparamagnetic iron oxide particle (VSOP) labeling on viability, stemness, and neuronal differentiation potential of primary human adult neural stem cells (haNSCs). Cytoplasmic VSOP incorporation massively reduced the transverse relaxation time T2, an important parameter determining MR contrast. Cells retained cytoplasmic label for at least a month, indicating stable incorporation, a necessity for long-term imaging. Using a clinical 3T MRI, 1 × 10^3^ haNSCs were visualized upon injection in a gel phantom, but detection limit was much lower (5 × 10^4^ cells) in layer phantoms and using an imaging protocol feasible in a clinical scenario. Transcriptional analysis and fluorescence immunocytochemistry did not reveal a detrimental impact of VSOP labeling on important parameters of cellular physiology with cellular viability, stemness and neuronal differentiation potential remaining unaffected. This represents a pivotal prerequisite with respect to clinical application of this method.

## Introduction

Stem cell transplantation represents one of the most promising strategies for the restoration of lost cells or tissue including their functions, or at least for delaying the pathogenic progress in neurodegenerative diseases (Janowski et al., 2015). The question whether adult or embryonic stem cells are best suited for clinical applications remains open (Modo et al., 2002; Dietrich and Kempermann, 2006), yet adult stem cells show reduced probability of teratoma formation, and thereby represent a promising candidate for clinical translation (Dietrich and Kempermann, 2006; Bellenchi et al., 2013). In humans, adult neural stem cells are present in the subventricular zone and in the subgranular zone of the hippocampal dentate gyrus (Zimmer et al., 1995; Stroh et al., 2005, 2009). These cells can be isolated from patients with intractable temporal lobe epilepsy subjected to neurosurgical intervention (Hoehn et al., 2002). Stem cells tracking and long term monitoring is demanded for thorough development of safe and efficient transplantation protocols and to further our understanding about therapeutic modes of action in both the preclinical and clinical setting.

To date, magnetic resonance imaging (MRI) of magnetically labeled stem cells represents the only clinically applicable imaging method for highly sensitive, non-invasive detection and long-term tracking of transplanted stem cells over extended periods of time (Modo et al., 2002; Stroh et al., 2005, 2009). Citrate-coated very small superparamagnetic iron oxide particles (VSOP) are widely used for pre-transplantation stem cell labeling. These particles are incorporated via endocytosis, aggregate and ultimately get stored in intracytoplasmic vesicles (Zimmer et al., 1995; Stroh et al., 2005). Incorporation of VSOP can be enhanced by lipofection to enhance the percentage of strongly labeled cells (Hoehn et al., 2002). Superparamagnetic iron oxide particles also seem to be biodegradable (Weissleder et al., 1989) and can even be utilized by the cells in iron metabolism pathways (Pouliquen et al., 1991).

However, the incorporation of iron oxide particles may lead to distinct changes in stem cell physiology (Stroh et al., 2004; Guzman et al., 2007; Focke et al., 2008; Neri et al., 2008). Moreover, superparamagnetic iron oxide/poly-L-lysine-labeling of a mouse neural stem cell line was reported to induce changes in expression of genes responsible for iron homeostasis, despite apoptotic pathways were not upregulated in that particular study (Walczak et al., 2006). Further, iron oxide particles have been shown to cause transient oxidative stress, closely linked to the free iron during incubation (Stroh et al., 2004), and most likely due to degradation and release of ferric ions into the acidic endolysosomal compartments (Soenen et al., 2011). Thus, evaluating the safety and impact of VSOP labeling on the differentiation potential of highly sensitive human adult stem cells is an important prerequisite for clinical translation (Gupta et al., 2007). Finally, the intended labeling protocol needs to be tailored to the respective stem cell population to ensure an acceptable balance between labeling intensity/efficacy and safety (Boltze et al., 2015).

Our study investigates describe the long term efficacy of human adult human neural stem cell (haNSC) VSOP labeling and its impact on viability, expansion, and neuronal differentiation potential for the first time. We analyzed both non-differentiated haNSCs as well as mature haNSCs-derived neurons, with murine embryonic stem cells (mESC) serving as cellular controls. We also assessed cell detection limit *in vitro* MRI with different modalities including one being close to a potential clinical application.

## Results

### Safety of Cell Labeling With VSOP

**FIGURE 1:**
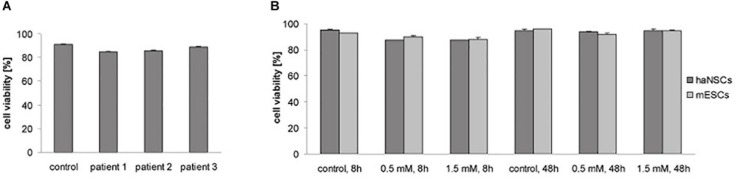
Cell viability of magnetically labeled haNSCs and mESCs (*n* = 3 with 3 technical replicates each). **(A)** Trypan blue exclusion test showed no significant differences in viability of three different patient samples (labeling with 0.5 mM). **(B)** Trypan blue exclusion test 8 and 48 h after labeling showed no decrease in cell viability due to the labeling procedure.

To assess safety of haNSC preparation, cryopreservation, and labeling (0.5 mM), or to detect any donor-dependent differences, cell viability was tested in the first step. No significant donor-dependent differences in cell viability between cells which underwent the labeling procedure with 0.5 mM (85–89%) and non-labeled control cells (91%, sample pooled from all patients) could be detected 1 day after labeling (Figure 1A). All samples could be included in onward experiments according to preset viability criteria (>80%). Next, cell viability of haNSCs and mESCs was compared 8 and 48 h after labeling with 0.5 and 1.5 mM VSOP, respectively (Figure 1B). Again, no significant differences in haNSCs viability between non-labeled control cells (95%), as well as 8 (87.5%) and 48 h (94.5%) after labeling became apparent. Viability of mESCs decreased slightly to 89% at 8 h and to 93.5% at 48 h after labeling. No viability differences were observed between 0.5 and 1.5 mM VSOP concentration.

### Efficacy of Magnetic Cell Labeling

**FIGURE 2:**
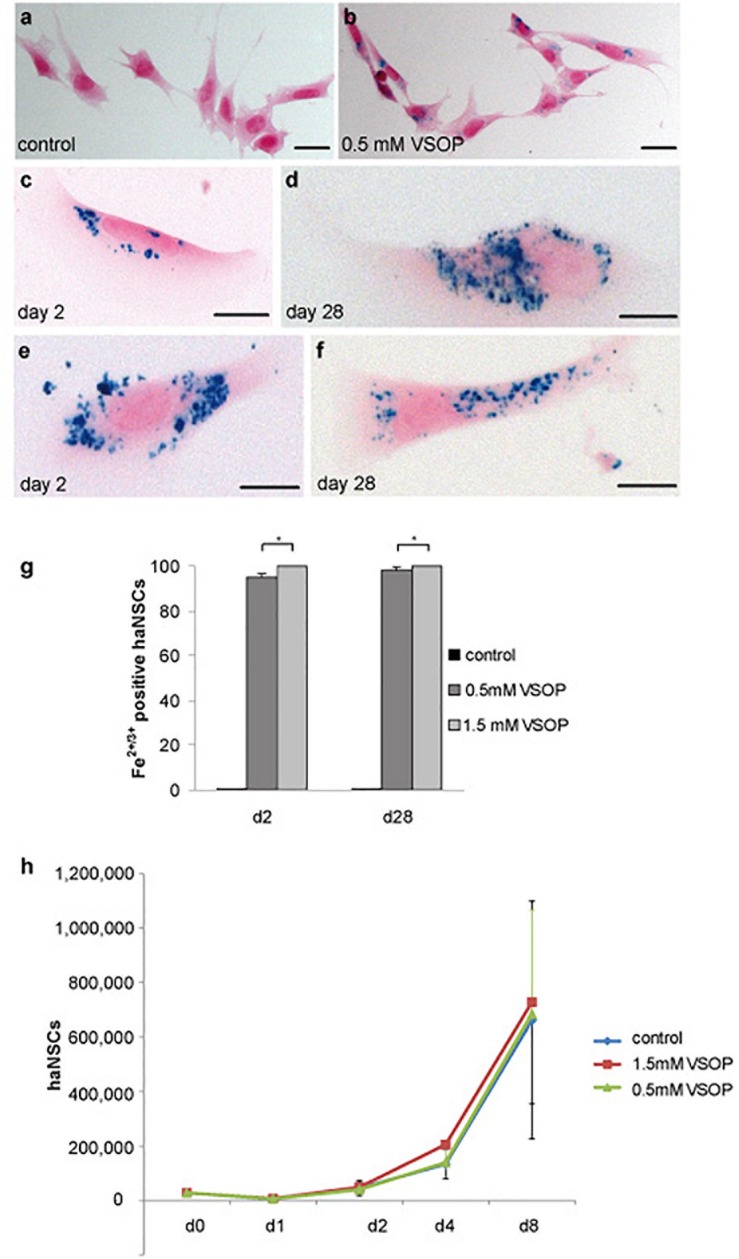
Cytological analysis of magnetically labeled haNSCs (*n* = 3 with 3 technical replicates each). **(a)** Unlabeled control cells and **(b)** intracytoplasmic VSOP uptake by haNSCs following incubation with 0.5 mM VSOP. **(c–f)** Cells were fixed with 4% phosphate-buffered saline-buffered paraformaldehyde and intracellular iron was visualized using Prussian blue staining on day 2 as shown in **(c,e)** and on day 28 as provided in **(d,f)** after labeling. **(g)** Cell counting revealed that labeling efficacy at any time point could not be enhanced significantly by lipofection. **(h)** Proliferation analysis of VSOP-labeled haNSC (1.5 mM) revealed no statistically significant difference in the proliferation abilities of unlabeled haNSC and haNSCs labeled with 0.5 mM VSOP, respectively. Scale bars in **(a–f)** represent 10 μm. ^∗^*p* < 0.01.

Incubation of haNSCs with 0.5 mM VSOP alone (“simple”) and additional lipofection resulted in a substantial uptake of magnetic label (Figure 2). Prussian blue staining revealed a homogenous ferric ion distribution in the cytoplasm, excluding the nuclei (Figures 2a,b). Prussian blue signals remained unchanged from day 2 to day 28 post simple incubation (Figures 2c,d), indicating a stable vesicular incorporation of VSOP for at least 1 month. This ratio did not differ significantly between day 2 and day 28. No apparent increase in iron-oxide particle uptake was observed upon visual inspection in lipofected cells (Figures 2e,f), which was confirmed by counting labeled cells. Overall, 96–100% of haNSCs were labeled. Labeling efficacy could not be improved significantly at any time point by additional lipofection (+L) (Figure 2g), so lipofection was omitted in all further experiments.

### Proliferation Assays

We next conducted a proliferation assay of labeled and non-labeled haNSCs. Over the course of 8 days, an exponential increase in cell number could be observed, yet, notably, no significant difference could be detected between the proliferation curves on non-labeled and haNSCs incubated with 0.5 and 1.5 mM VSOP (Figure 2h).

### Magnetic Resonance Properties

**FIGURE 3:**
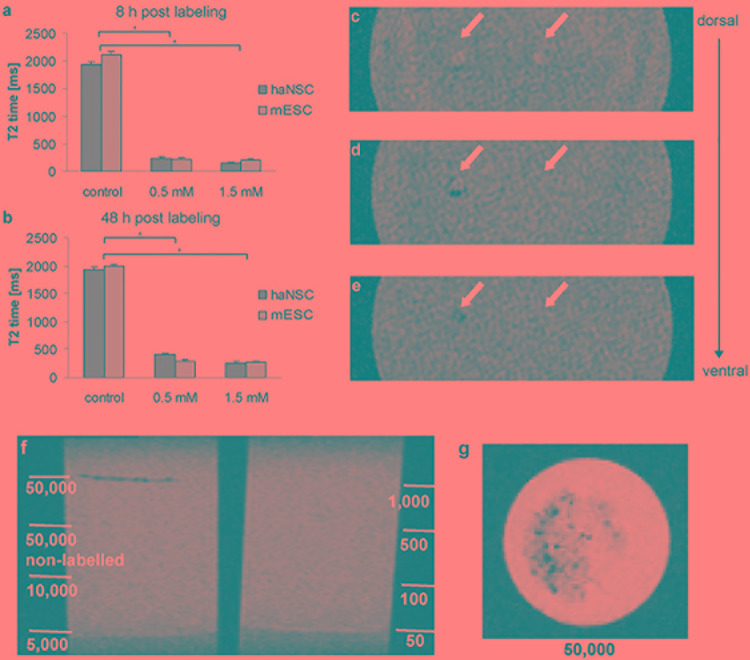
NMR relaxometry and gradient echo MR images of gel phantoms containing haNSCs (*n* = 3 or more). **(a,b)** Efficiency of VSOP-labeling was determined after 8 **(a)** and 48 h **(b)** using NMR relaxometry. All data were analyzed and presented as mean ± SEM. Differences were considered significant at *P* < 0.01. **(c)** Coronal section at gel surface showing two needle tracks as hyperintense signal change. 1 × 10^3^ VSOP labeled haNSCs were injected on the left side; on the right side 1 × 10^3^ unlabeled control haNSCs were injected. **(d)** Slice 4.5 mm ventral of **(c)**. In the left injection track an area of signal loss can be visualized, due to clusters of magnetically labeled haNSCs. No signal change in the right injection track was observed. **(e)** Slice 5 mm ventral of **(c)**. The left injection track again shows an area of signal loss, no signal change in the right injection track. **(f,g)** layer phantoms investigated in 3T SWI MRI. Only 5 × 10^4^ labeled haNSCs were detectable with an imaging protocol being short enough (41:00 min) to be applicable in a hypothetical clinical scenario. ^∗^*p* < 0.01.

Next, the impact of 0.5 and 1.5 mM VSOP-labeling of both stem cell populations on magnetic resonance properties was determined after 8 and 48 h via NMR relaxometry (Figures 3a,b). Unlabeled cells had a T2 time of 2110 ms (mESCs) and 1948 ms (haNSCs), revealing no significant difference between both protocols. In contrast, VSOP-labeling with 0.5 mM and 8 h incubation time led to a significant reduction of T2 time to 10% of control values (mESCs: 218 ms, haNSCs: 227 ms, Figure 3a, *P* < 0.01). 48 h after incubation, T2 time ranged at 15% of control values (mESCs: 298 ms, haNSCs: 408 ms, Figure 3b). Further increasing VSOP concentrations to 1.5 mM resulted in an additional reduction of average T2 time which, however, was not statistically significant from that after 0.5 mM incubation.

### *In vitro* MR Imaging

To assess clinical applicability of the labeling protocol, gradient echo MR images of gel phantoms with injected cells were acquired (Figures 3c,g). On the T2^∗^-weighted images two needle tracks on the gel surface can be delineated as hyperintense signal changes (Figure 3c) in injection phantoms. On the left side of the phantom 1 × 10^3^ magnetically labeled haNSCs were injected, whereas 1 × 10^3^ unlabeled control haNSCs where placed on the right side. The T2^∗^-weighted images clearly showed hyperintense signal changes only in the left injection track deposit of 1 × 10^3^ labeled haNSCs (Figures 3d,e). Cell detectability was lower in layer phantoms imaged at 3T SWI with a protocol that could be applied on hypothetical patient. Only 5 × 10^4^ labeled cells were detectable with the applied imaging protocol (Figures 3f,g).

### Characterization of Non-differentiated VSOP Labeled Stem Cells

**FIGURE 4:**
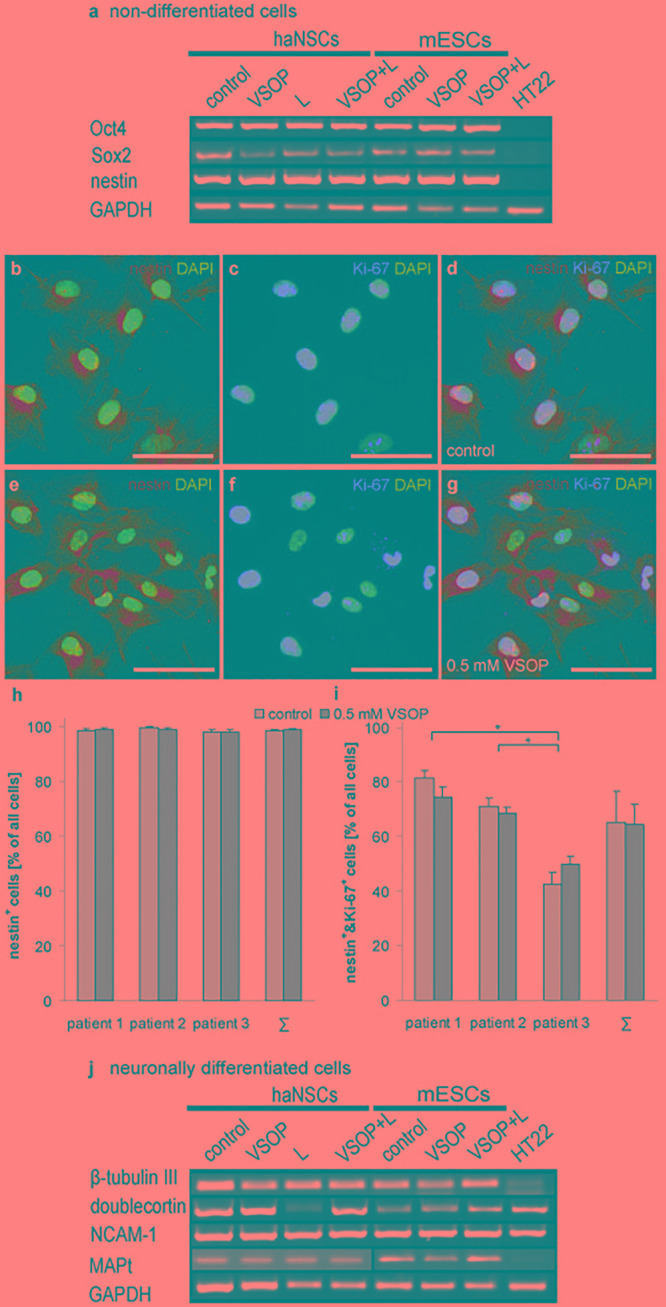
Transcriptional and immunohistochemical analysis of stemness and neuronal differentiation (*n* = 6 to 10). **(a)** VSOP-labeling and lipofection of non-differentiated stem cells; RT-PCR analysis of pluripotency markers revealed no impact on stemness, and transcribed nestin. 99% of haNSCs expressed the intermediate filament protein nestin, and 65% co-express the nuclear proliferation marker Ki-67. No significant difference between control cells and VSOP labeled haNSCs could be detected. **(b–d)** show unlabeled control cells, and **(e–g)** give the VSOP labeled pendant. **(h)** Nestin expression of haNSCs from three different patients was compared, showing that there is neither a significant difference between patients nor between control cells or labeled cells. **(i)** 65% of haNSCs co-express nestin and Ki-67, but significant differences between the three patients become apparent. **(j)** Subsequent neuronal differentiation (*n* = 6 to 10) resulted in a neuronal phenotype transcribing β-tubulin III, doublecortin, N-CAM1, and MAP-2. Mature mouse hippocampal cells HT22 served as a negative control for pluripotency markers and as a positive control for mature neuronal markers. Scale bars represent 50 μm. ^∗^*p* < 0.01.

Cells were magnetically labeled and subsequently analyzed by RT-PCR and Western Blotting (Figure 4a). Non-labeled haNSCs and mESCs served as cellular controls. After culturing in the appropriate expansion media, all cells except the HT22 expressed the mRNA for the stemness markers Oct4 and Sox2, and the neural progenitor marker nestin. haNSCs and mESCs loaded with 0.5 mM VSOP (VSOP) or VSOP plus lipofection agent (VSOP + L) revealed the same mRNA expression pattern. No difference between stem cells incubated with (L) and without lipofectin became apparent.

Analysis of haNSCs using fluorescent immunocytochemistry showed that 99% of cells express the intermediate filament protein nestin (Figure 4b). 65% co-expressed the nuclear proliferation marker Ki-67 (Figures 4c,d). 99% of haNSCs, which underwent the VSOP-labeling procedure and subsequently had a high cytoplasmatic iron loading, expressed nestin while 64% of cells were found positive for Ki-67 (Figures 4e–g). Substantial differences between control cells and VSOP labeled haNSCs from all three donors could not be observed (Figure 4h). Analysis of nestin and Ki-67 co-expression revealed no impact of VSOP labeling (Figure 4i). However, we found a significant effect on relative numbers of cells co-expressing nestin and Ki-67 in the three patients (*P* < 0.01).

### Differentiation Potential of VSOP-Labeled Stem Cells

Figure 4j illustrates the analysis of stem cells that were magnetically labeled and subsequently neuronally differentiated over 15 days. Non-labeled, but neuronally differentiated haNSCs and mESCs served as controls. To characterize the transition from neural progenitors to mature neurons, mRNA expression levels of β-tubulin III, doublecortin (DCX), neural cell adhesion molecule 1 (NCAM-1) as well as microtubule associated phosphoprotein 2 (MAP-2) were investigated. RT-PCR analysis indicated neural progenitors and immature neurons in control cell populations as well as in magnetically loaded cell populations. No differences in expression patterns were observed.

## Discussion

In this study, we were able to show that labeling of haNSC with iron-oxide-particles, required for a high-resolution MR Imaging, is both safe and efficient. Labeling efficiency using 0.5 mM VSOP was found up to 100% and resulted in stable cell labeling for at least 1 month. Trypan blue exclusion tests at different time points and comparison of different patient samples demonstrated that VSOP-labeling had no effect on overall haNSCs viability. Using clinical 3T MRI, we visualized 1 × 10^3^ labeled cells in an injection phantom, modeling the *in vivo* situation under optimal imaging conditions. Moreover, 3T SWI MRI of layer phantoms with layers containing up to 5 × 10^4^ labeled cells was performed, applying an imaging protocol taking 41:00 min. This is short enough to be applied in a hypothetical clinical scenario, but on the upper limit what could be performed in critically ill patients. The protocol settings were chosen on optimal detectability in VSOP-labeled mesenchymal stem cells (Stroh et al., 2011). The analysis of Oct4 and Sox2 mRNA-expression in addition to the quantitative fluorescence immunocytochemistry analysis of Ki-67 and nestin co-expression as stem cell markers and indicators of proliferation activity showed no impact of VSOP labeling, but significant differences in haNSC samples from different donors. Furthermore, neuronal differentiation potential and neuronal marker expression were not affected by the labeling procedure.

Prussian blue staining resulted in a homogenous ferric ion distribution in the cytoplasm, but not in the nucleus. This is in line with previous findings from our group, showing a direct relation between VSOP incubation, intracellular iron load as measured by atomic absorption spectroscopy, and T2 time reduction (Stroh et al., 2005). Prussian blue staining was found stable after 48 h and 28 days, indicating a transfer of VSOP onto daughter cells during cytokinesis for proliferating cells. Our data indicate no difference in labeling efficiency and stability between incubation with VSOP and additional lipofection. One explanation of the efficient incorporation with VSOP alone could be the small diameter and the negative surface charge of the citrate coating, already allowing efficient uptake without additional lipofection. Albeit lipofection did not raise a safety concern in our study, off-target effects and immune response caused by transfection reagents have been described (Bauer et al., 2006). Thus, sufficient labeling with VSOP but omitting lipofection may represent a clear advantage for subsequent *in vivo* and clinical applications.

It is known that citrate coating may interact with the extracellular domain of integrins and triggers their activation and subsequently intracellular signaling cascades (Miller et al., 2010). Furthermore, VSOP can induce increased levels of transferrin receptor-1. VSOP incubation can also result in a fast increase and high level of reactive oxygen species (ROS), most likely due to degradation and release of ferric ions into the acidic endolysosomal compartments (Soenen et al., 2011). Although no detrimental influence has been shown in the present study, the overall impact of these potential alterations should be carefully controlled after *in vivo* transplantation. While measures of T2 relaxation times provide an indication of the cellular iron uptake, a direct measure of iron content, as conducted in previous studies of ours using the same iron-oxide particles, yet in different cells, would be desirable (Stroh et al., 2005, 2009). We were not able to conduct these assays due to the scarcity of the cellular material. Also, iron-oxide particles have shown to induce oxidative stress (Stroh et al., 2004), potentially leading to oxidative damage to proteins, lipids and even DNA, also at low concentrations of the label (Novotna et al., 2012). Viability assays, as conducted here, might not be sensitive toward these molecular changes, but again, we were not able to conduct these assays due to the limited availability of the cells.

Potential effects of iron oxide on the neuronal differentiation potential of haNSC and mESC, were analyzed on the gene transcription level. The present data indicates that VSOP-labeled haNSCs and mESCs can be expanded without affecting their potential for both neurogenesis, as represented by the pluripotency markers Sox2 (Suh et al., 2007) and Oct4 (Nichols et al., 1998), or their ability for neuronal differentiation. Magnetic labeling had also no impact on mRNA transcription of nestin and Ki-67 expression even after 14 days in culture. In this study, we used iron-oxide nanoparticles, which are presumably taken up by the cells by endocytosis (Stroh et al., 2004). This means, that in contrast to genetically encoded labels, the concentration of particles in the cells will diminish over time, both due to cell proliferation, and also due to particle degradation. This limits the time window, in which these cells will be detectable by MRI. What is more, we provided data on RNA level on the differentiation potential of these labeled cells, but this does not prove a functional stable differentiation into a given neuronal subtype. For that, additional assays on protein level and ultimately electrophysiological evidence need to be conducted (Stroh et al., 2011).

1 × 10^3^ VSOP-labeled haNSC were easily identified in a gel phantom, reliably modeling the situation upon stereotactic transplantation *in vivo* (Stroh et al., 2005). This detection range is more than sufficient to monitor cell migration in clinical scenarios where 20 to 300 × 10^3^ or even more cells are transplanted (Kondziolka et al., 2004; Stover et al., 2005). Nevertheless, the detection limit is rather high compared to other studies that repeatedly shown single cell detectability. A direct comparison is difficult though. For instance, some studies employed ultra-high field strengths (11.7T) and imaging protocols optimized for that purpose (Hinds et al., 2003). Others used long imaging times and different cell types such MDA-MB-231BR breast cancer cells or J774 macrophages (Heyn et al., 2006). Both cell types are much larger (up to 25 μm diameter and up to 100 μm in length for MDA-MB-231BR) than neural stem cells and therefore can store much higher amounts of VSOP, while macrophages are particularly efficient in uptaking VSOPs. We also had to apply relatively long scanning times to visualize 1 × 10^3^ labeled cells. Moreover, ultra long-term *in vivo* imaging at 3T may necessitate higher cell concentrations as cell loss or dilution of intracellular iron content due to *in vivo* proliferation may reduce the overall signal obtained. It should be noted though that cell detectability is different in gel phantom and *in vivo* imaging, the latter commonly associated with higher detection limits.

On the other hand, 3T SWI with a short-term protocol optimized for cell detectability was only able to detect 5 × 10^4^ cells in a layer phantom, modeling wide-spread cell distribution as can be observed after systemic transplantation or migration. Apart from the shorter imaging time and the lower number of averages, the higher detection limit might have two potential reasons. First, layer phantoms represent the cell distribution seen after systemic (intraarteriell or intravenous) cell injection. Systemic cell injection is believed to be more feasible for clinical applications and is therefore used predominantly in ongoing clinical trials (Cui et al., 2019). The density of cells is lower than in injection phantoms that represent the situation after stereotactic cell implantation procedures, being reserved for a targeted approach requiring local cell deposits. Second, the imaging protocol applied for layer phantom imaging was developed for mesenchymal stem cells which are larger than haNSCs and might therefore incorporate more VSOP leading to better detectability. 7T MRI, which is being introduced into clinical imaging, and allowing for detection of small local concentrations of labeled cells while the feature of VSOP to further reduce the longitudinal (T1) and transverse (T2) relaxation time may provide much better cell detectability at high filed strengths in future applications (Klug et al., 2010).

In summary, our study strongly suggests that in the clinical setting, cell tracking using magnetic VSOP labeling represents a safe method, capable of addressing three key issues vital for effective transplantation strategies: location, migration, and viability.

## Materials and Methods

### Ethics Statement

Human adult neural stem cells were obtained from patients with temporal lobe epilepsy submitted to epilepsy surgery. Informed and written consent was given for additional scientific investigations approved by the local ethics committee of the University of Erlangen, in accordance with the Declaration of Helsinki.

### Human Adult Neural Stem Cell and Murine Embryonic Stem Cell Cultures

Composition of expansion and differentiation media is given in Table 1. After surgical en bloc resection, the dentate gyrus was micro-dissected and dissociated mechanically followed by enzymatic digestion as described previously (Hoehn et al., 2002). Of the obtained cells, haNSC from passage 5 to 11 were cryopreserved, shipped to the primary investigation site and used for all experiments. Cells were plated on poly-L-ornithine (250 μg/mL)-/laminin (15 μg/mL, both Sigma-Aldrich, Munich, Germany)-coated cell culture dishes (Sarstedt, Nuembrecht, Germany) and grown in expansion medium at 37°C and 5% CO_2_. Medium was changed every second day. Cells were detached with accutase (PAA, Cölbe, Germany) at 80% confluence, and split. Culturing over at least five passages selected for proliferating cells.

**TABLE 1:** Expansion and differentiation media.

**haNSC**		**mESC**				
**Basic cell culture medium**				**mESC-derived nestin+ neural progenitors**		
Knockout-DMEM and Ham’s F12, 5% knockout serum replacement, 2 mM L-glutamine, 1% penicillin-streptomycin, 1% MEM non-essential amino acids, and 1% N2 supplement (all Invitrogen, Karlsruhe, Germany)						

**Expansion**	**Differentiation**	**Expansion**	**Differentiation**	**Selection medium**	**Expansion**	**Differentiation**
**medium**	**medium**	**medium**	**medium**	**(from mESC)**	**medium**	**medium**
Basic cell culture medium	Basic cell culture medium	Basic cell culture medium	Basic cell culture medium	Knockout-DMEM and Ham’s F12	haNSC/mESC basic cell culture medium	Expansion medium
+10 ng/mL basic fibroblast growth factor (bFGF, Invitrogen)	+2% B-27 supplement	+0.1 mM 2-mercaptoethanol	Embryoid body formation in bacterial dishes for 6 days, final differentiation in gelatine-coated dishes	+1 % ITS supplement (insulin-transferrin-selenium, Invitrogen)	+10% knockout serum replacement	- N2 supplement
+10 ng/mL epidermal growth factor (EGF, Invitrogen)	+200 ng/mL sonic hedgehog (SHH)	+15 ng/mL LIF			+10 ng/mL bFGF	- bFGF
+2.5 μg/mL bovine pituitary extract (BPE, Sigma-Aldrich)	+100 ng/mL fibroblast growth factor-8 (FGF-8)	On mouse fibroblasts (CRL-1503, Sigma)				+20 ng/mL nerve growth factor (NGF) (Invitrogen)
+15 ng/mL leukemia inhibitory factor (LIF, Sigma-Aldrich)	+2 mM ascorbic acid					+1× B-27 supplement (Invitrogen)
+2 μg/mL heparin						

Previously cryopreserved mouse embryonic stem cells (mESCs, CRL-1934, ATCC, Manassas, United States) were cultured in expansion medium (Table 1) at 37°C and 5% CO_2_ on mouse fibroblasts (CRL-1503, ATCC, Manassas, United States) inactivated by mitomycin C (Sigma-Aldrich). Stem cells were separated from the fibroblast layer at 60% confluence by detaching them with accutase, and transferred to gelatin-coated cell culture dishes with daily medium changes. mESCs were detached and transferred every other day. A fibroblast-free mESCs culture was obtained after three passages.

### Magnetic Cell Labeling and Viability Assessment

Sterile VSOP (C200, Ferropharm, Teltow, Germany), consisting of a 5nm iron oxide core coated with monomer citrate, resulting in a hydrodynamic diameter of 11 nm and a negative charge were used for labeling at 0.5 and 1.5 mM with or without additional lipofection agent (lipofectin, Invitrogen) for 4 h as described previously (Gupta et al., 2007). These molarities were chosen based on previous findings showing that VSOP labeling causes transient oxidative stress or even irreversible cell damage (Stroh et al., 2004, 2009). Transient oxidative stress is already observed at 1.5 mM, and higher incubation molarities such as 3.0 or 6.0 mM cause more intensive stress or even increase apoptosis and necrosis. In turn, higher VSOP incubation molarities not necessarily shortens T2 relaxation times or increases detectability, particularly in smaller cells (Stroh et al., 2009).

Control specimens were incubated in Opti-MEM I for 4 h instead. First, potential donor-specific viability differences were assessed 20 h after labeling with 0.5 mM VSOP. Only cells samples with >80% viability after labeling were defined acceptable for further investigation. Next, cell viability in relation to incubation molarities was assessed 8 and 48 h after VSOP incubation by the Trypan Blue (Sigma-Aldrich) exclusion test on 10 randomly selected regions per coverslip.

### Labeling Efficacy Assessment and haNSC Proliferation Experiments

Uptake of VSOP was evaluated using Prussian blue staining 48 h and 28 days after VSOP labeling by phase contrast microscopy of 10 regions per coverslip. The relative labeling efficacy was displayed as a ratio of Prussian blue positive cells to all cells identified by nuclear counterstaining.

After labeling with 0.5 μM or 1.5 μM VSOP (see above), haNSCs were seeded on 12-well-plates covered with poly-L-ornithine-covered (Sigma-Aldrich) at a density of 7,500 cells/cm^2^ (28,500 cells/well) on expansion medium. Unlabeled haNSCs served as controls. Proliferation of haNSCs was monitored over 8 days, and cell culture medium was exchanged every second day. Cell numbers were assessed on days 1, 2, 4, and 8 in three wells for each VSOP concentration and on each day. Experiments were performed with using a total of six haNSC lines derived from three different patients (*n* = 3).

### NMR Relaxometry

Further, nuclear magnetic resonance (NMR) relaxometry was performed to determine the T2 time (transverse relaxation time). 9 × 10^5^ cells were suspended in 3 mL PBS and measured in a NMR-Relaxometer Minispec 0.47T/20MHz (Bruker, Ettlingen, Germany). Data analysis was performed using the ORIGIN Pro 8G software (OriginLab Corporation, Northampton, MA, United States). All samples have been analyzed in three independent experiments.

### Clinical MR Imaging at 3 Tesla

Detectability of VSOP labeled haNSCs was assessed by *in vitro* MRI. Two different types of agarose gel phantoms, cell injection and cell layer phantoms, were used. For injection phantoms, 1 × 10^3^ magnetically labeled haNSCs (1.5 μM VSOP) were injected into air bubble-free gel phantoms as described previously (Soenen et al., 2011). Briefly, 1 × 10^3^ magnetically labeled haNSCs were injected on one side of the phantom, and 1 × 10^3^ non-labeled haNSCs on the other.

Injection phantom MRI was performed on a clinical 3 Tesla scanner (Achieva 3T, Philips Medical Systems, Best, Netherlands) under conditions optimized for cell imaging. A 3T solenoid rat coil (RX SN 1116, Philips) with a coil diameter of 55 mm was used. 2D gradient echo sequences were applied, with TE/TR 13.8/50 ms, a flip angle of the excitation pulse of 15°, yielding images that were T2^∗^ (effective transverse relaxation time) weighted. Both a coronal as well as a sagittal data set was acquired covering the entire gel phantom. The slice thickness was 0.3 mm, the interslice distance 0.15 mm, with a 20 mm field of view, and a 288 × 288 matrix, resulting in an inplane resolution of 69 μm and a voxel size of 69 × 69 × 300 μm^3^.

Layer gel phantoms were produced as described elsewhere (Lobsien et al., 2013). Two phantoms were produced, each containing four layers in which unlabeled or VSOP-labeled haNSCs (1.5 μM) were embedded. Layer phantom 1 contained layers with 5 × 10, 1 × 10^2^, 5 × 10^2^, and 1 × 10^3^ VSOP-labeled haNSCs, respectively. Layer phantom 2 contained layers with 5 × 10^3^, 1 × 10^4^, and 5 × 10^4^ VSOP-labeled haNSCs, as well as a layer containing 5 × 10^4^ unlabeled haNSCs.

Layer phantom MRI was performed on a clinical 3 Tesla Scanner (Magnetom Trio, Siemens, Erlangen, Germany) equipped with a standard 8ch knee coil. The imaging protocol was not particularly optimized for cellular imaging but rather to reflect a clinical situation. 3D Susepctibility-weighted images (SWI) were obtained with TE/TR 20/60 ms at a flip-angle FA of 15°. A voxel size of 560 μm^3^ × 490 μm^3^ × 250 μm^3^ was achieved. Acquisition time TA was 41:00 min. SWI sequences were processed by vendor-specific software of the MRI scanner (Syngo B15, Siemens, Erlangen, Germany) and analyzed without further post-processing.

### RT-PCR Analysis

Cells were harvested before and after magnetic labeling, and neuronal differentiation (see below), respectively. RNA species >200 nt were extracted using Mini RNeasy kit. cDNA synthesis with integrated removal of genomic DNA contamination was performed on 1 μg RNA using QuantiTect Reverse Transkription kit (both Qiagen, Hilden, Germany). PCR was performed on 2 μl of cDNA using Taq-DNA-Polymerase all inclusive kit (Peqlab, Erlangen, Germany). Table 2 shows the primer sequences used in this experiment. All samples were analyzed in at least three independent experiments. Mature mouse hippocampal cells HT22 were used as a negative control for pluripotency and neural progenitor markers.

**TABLE 2:** List of primers.

**Gene**	**Primer forward**	**Primer reverse**
Oct4	5′- CTCCTGAAGCAGA AGAGGATCAC-3′	5′-CTTCTGGCGCCGG TTACAGAACCA-3′
Sox2	5′-TGCAGTACAACT CCATGACCA-3′	5′-GTGCTGGGACATGT GAAGTCG-3′
nestin	5′-CAGCGTTGGAACAG AGGTTG-3′	5′-GCTGGCACAGG TGTCTCAAG-3′
β_3_-tubulin	5′-GCAAGGCCTTCC TGCACT-3′	5′-GCGTCCTGGTACT GCTGGTA-3′
DCX	5′-GACAGCCCACTCT TTTGAGC-3′	5′-GCGTAGAGATGGGA GACTGC-3′
NCAM1	5′-TATCCCAGTGCCA CGATCTC-3′	5′-TGGCTTCCTTGGC ATCATAC-3′
MAP-2	5′-GGTGGCAAGGTGC AGATAAT-3′	5′-CTTTGGCATTCTCC CTGAAG-3′
(*mus musculus*)		
MAP-2	5′-GTGGGGGTTGTC ACAGAGG-3′	5′-GCTCTCCCAGCG GCAAGG-3′
*(homo sapiens)*		
GAPDH	5′-CAACGAATTTGGC TACAGCA-3′	5′-AGGGGTCTACATG GCAACTG-3′

### Fluorescence Immunocytochemistry and Characterization

For characterization of cultured haNSCs and for determining effects of magnetic labeling on stemness, cells were fixed in PBS-buffered paraformaldehyde (4%). Processing, immunocytochemistry and quantitative evaluation were performed as described elsewhere (Heyn et al., 2006). Mouse-anti-Ki67 (Dako, 1:100, proliferation marker), and rabbit-anti-nestin (Millipore, 1:200, neural progenitor cell marker) were used as primary antibodies, and Alexa Fluor 555 or 488 (both Invitrogen, 1:100) served as secondary antibodies. Nuclei were labeled by 4′,6-Diamidino-2-phenylindole dihydrochloride (DAPI, Sigma-Aldrich, 1.1000). For analysis, 10 randomly selected regions per coverslip were counted. Percentage was determined as the number of nestin^+^, Ki-67^+^, or Ki-67^+^/nestin^+^ cells compared to total cells identified by nuclear DAPI counterstaining.

### Neuronal Differentiation of haNSCs and mESC

The possible interference of VSOP-labeling or lipofectin on neuronal differentiation was also assessed. haNSC were differentiated for 15 days in haNSC differentiation medium (Table 1), which was changed every second day. Six independent experiments were conducted, containing at least three technical replicates each.

mESCs-differentiation was performed as described elsewhere (Bibel et al., 2004) with some modifications. For embryoid body formation expanded mESCs were transferred into bacterial dishes and LIF was removed. After 6 days cells had aggregated and formed embryoid bodies, which were carefully transferred to gelatine-coated dishes. During the following 2–3 days, the heterogeneous bodies adhered and migrating cells started to form a monolayer. Neural progenitor cells were selected for 3 days and then expanded in neural progenitor expansion medium (Table 1) for 10 days. RT-PCR analysis and Prussian blue staining were performed as described above. Ten independent experiments were conducted.

### Statistical Analysis

Statistical analysis was conducted using SPSS (Chicago, IL, United States) software. First, data sets from all conditions were tested for normal distribution using the parameter free one-sample Kolmogorov-Smirnov test. In cases where normal distribution of data was confirmed, the parametric two-tailed Student’s *t*-test was employed to compare means. *P*-values <0.01 were considered statistically significant. All data are presented as mean ± standard error of the mean (SEM).

## Data Availability Statement

The datasets generated for this study are available on request to the corresponding author.

## Ethics Statement

Human adult neural stem cells were obtained from patients with temporal lobe epilepsy submitted to epilepsy surgery. Informed and written consent was given for additional scientific investigations approved by the local ethics committee of the University of Erlangen, in accordance with the Declaration of Helsinki.

## Author Contributions

AS, JK, IB, JS, CZ, and JB: conception and design. AS, JK, RC, AD, WF, and SZ: collection of data. AF and DL: collection of data, and data analysis and interpretation. AS, JK, and JB: data analysis and interpretation, and manuscript writing. All authors have approved the final manuscript.

## Conflict of Interest

The authors declare that the research was conducted in the absence of any commercial or financial relationships that could be construed as a potential conflict of interest.
